# Stratification of coronary artery disease patients for revascularization procedure based on estimating adverse effects

**DOI:** 10.1186/s12911-015-0131-0

**Published:** 2015-02-14

**Authors:** Sebastian Pölsterl, Maneesh Singh, Amin Katouzian, Nassir Navab, Adnan Kastrati, Lance Ladic, Ali Kamen

**Affiliations:** 1 Computer Aided Medical Procedures, Technische Universität München, Boltzmannstr. 3, 85748 Garching b. München, Germany; 2 Siemens Corporation, Corporate Technology, Imaging and Computer Vision, 755 College Rd E, Princeton, NJ USA; 3 Deutsches Herzzentrum and 1. Medizinische Klinik rechts der Isar, Technische Universität München, Lazarettstr. 36, 80636 München, Germany; 4 Siemens Healthcare Diagnostics, Strategic Innovation Group, 511 Benedict Ave, Tarrytown, NY USA

**Keywords:** Coronary artery disease, Decision making, Decision support systems, Restenosis, Stents, Coronary artery bypass

## Abstract

**Background:**

Percutaneous coronary intervention (PCI) is the most commonly performed treatment for coronary atherosclerosis. It is associated with a higher incidence of repeat revascularization procedures compared to coronary artery bypass grafting surgery. Recent results indicate that PCI is only cost-effective for a subset of patients. Estimating risks of treatment options would be an effort toward personalized treatment strategy for coronary atherosclerosis.

**Methods:**

In this paper, we propose to model clinical knowledge about the treatment of coronary atherosclerosis to identify patient-subgroup-specific classifiers to predict the risk of adverse events of different treatment options. We constructed one model for each patient subgroup to account for subgroup-specific interpretation and availability of features and hierarchically aggregated these models to cover the entire data. In addition, we deviated from the current clinical workflow only for patients with high probability of benefiting from an alternative treatment, as suggested by this model. Consequently, we devised a two-stage test with optimized negative and positive predictive values as the main indicators of performance. Our analysis was based on 2,377 patients that underwent PCI. Performance was compared with a conventional classification model and the existing clinical practice by estimating effectiveness, safety, and costs for different endpoints (6 month angiographic restenosis, 12 and 36 month hazardous events).

**Results:**

Compared to the current clinical practice, the proposed method achieved an estimated reduction in adverse effects by 25.0% (95% CI, 17.8 to 30.2) for hazardous events at 36 months and 31.2% (95% CI, 25.4 to 39.0) for hazardous events at 12 months. Estimated total savings per patient amounted to $693 and $794 at 12 and 36 months, respectively. The proposed subgroup-specific method outperformed conventional population wide regression: The median area under the receiver operating characteristic curve increased from 0.57 to 0.61 for prediction of angiographic restenosis and from 0.76 to 0.85 for prediction of hazardous events.

**Conclusions:**

The results of this study demonstrated the efficacy of deployment of bare-metal stents and coronary artery bypass grafting surgery for subsets of patients. This is one effort towards development of personalized treatment strategies for patients with coronary atherosclerosis that could significantly impact associated treatment costs.

**Electronic supplementary material:**

The online version of this article (doi:10.1186/s12911-015-0131-0) contains supplementary material, which is available to authorized users.

## Background

Treatment of coronary atherosclerosis due to accumulation of plaques throughout arteries often encompasses mainly two options: minimally invasive percutaneous coronary intervention (PCI) or open-chest coronary artery bypass grafting (CABG). The latter distinguishes itself from PCI by being a more elaborate procedure and by better long-term prognosis [1]. During PCI, the normal blood flow is established within the narrowed region (stenosis) by deploying either stents (small mesh tubes) or balloons that push away the atherosclerotic plaques toward the vessel wall. The two most commonly used types of stents are: bare-metal stents (BMS) and drug-eluting stents (DES).

Treatment of coronary occlusions with BMS implantation has shown to be associated with a high rate (20-30%) of restenosis (re-narrowing of the treated artery) [2-4], which is significantly lower (4-8%) with DES treatment [5,6]. However, at the same time a higher rate of late thrombosis (>1 year after initial treatment) is found to be associated with DES (5 events per 1000 patients compared to no events) [7,8]. Therefore, in contrast to bare-metal stents, drug-eluting stents have a lower risk of early restenosis, but require a prolonged dual antiplatelet therapy, which may lead to thrombosis in case of early discontinuation [9,10].

Overall, the results of multiple studies comparing outcomes by treatment type have shown that 1) the rate of myocardial infarction, death, stroke, and stent thrombosis is similar for BMS and DES, but that BMS has a higher rate of restenosis, and thus revascularization [11-14], and that 2) CABG is superior to DES with respect to major adverse cardiac or cerebrovascular events [1,14]. In addition to individual pros and cons, each treatment option is accompanied by different costs; initial treatment costs for CABG are higher than for PCI and initial DES treatment is more expensive than BMS.

Several studies showed that DES is superior to their BMS counterpart only for a subset of patients [15-18]. Amin et al. [19] quantified the benefits of both options. They showed that reducing the usage of DES by 50% for patients at low risk of target-vessel revascularization (TVR) could save up to $205 million per year in U.S. health care costs, while the overall rate of TVR was projected to increase by 0.5%. In response, Cavender and Ellis [20] pointed out several flaws in the analysis by Amin et al. [19]: 1) they used unbalanced data (the vast majority had been treated with DES), 2) they used TVR as a proxy for restenosis, and 3) that their model discriminated only modestly.

From a treatment cost perspective, multiple studies compared the cost-effectiveness of CABG and PCI for patients with multivessel coronary artery disease [21-25]. Varani et al. [22] concluded that the total treatment costs during the first year were lower with PCI employing DES, whereas the remaining studies determined that CABG was more cost-effective than PCI.

The SYNTAX^a^ score II is a tool to aid decision making between CABG and PCI. It augments the anatomical SYNTAX score with seven clinical variables, which are strongly associated with mortality in either the PCI or CABG setting in patients with complex coronary artery disease [26]. The authors concluded that they could accurately predict 4-year mortality of patients. However, their model is only applicable to patients with complex coronary artery disease. Similarly, the authors of [27] developed a decision support tool for CABG and PCI with BMS or DES to predict long-term mortality (5 and 10 years) under each treatment option. Although, their models are based on a general patient population, they did not provide quantitative results with respect to the predictive power of their models or the economic impact.

Our aim was to develop a model that captures the current clinical knowledge about risks and benefits of treatment options for atherosclerosis to recommend a more personalized treatment regime for patients. If risks can be predicted reliably, it would be possible to deviate from the existing clinical practice and replace predominant DES treatment with either BMS, for patients with low risk of future restenosis, or CABG for patients at high risk of death, myocardial infarction, or thrombosis. To achieve this, we formed two hierarchical models: the first is to assess the risks of BMS treatment and the second is to evaluate the risks of DES treatment. The subgroup-specific models allowed us to overcome the lack of available features for all patients. Furthermore, by including in vitro diagnostic biomarkers, we demonstrated a potential improvement on both the patient outcome and the estimated overall treatment costs.

### Clinical motivation

Our approach was motivated by two recent shifts in the clinical practice. First, although stenting predominately employs DES with particular focus on reducing restenosis rates, recent studies showed that about one third of patients in fact do not draw much benefit from DES [15]. Moreover, this specific patient subgroup might unnecessarily be exposed to a higher risk of thrombosis leading to major adverse events. Subsequently, for these patients the better treatment choice both clinically and financially could be BMS.

**Figure 1:**
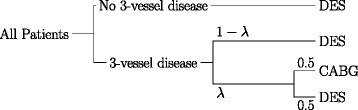
**Baseline treatment workflow.** Patients with no 3-vessel disease are treated with drug-eluting stents (DES). For the remaining patients we assumed that a fraction of them (*λ*) would be eligible for both DES and coronary artery bypass grafting (CABG); hence, we assumed that half of these patients are treated with DES and the other half with CABG.

Second, it has been shown that patients, who were traditionally in a grey area for eligibility of both DES and CABG (see Figure 1), could benefit from DES treatment. These include diabetic patients with mild to less severe coronary artery disease affecting multiple vessels [1]. For these patients, who are not particularly at high risk of thrombosis and hence major adverse events with DES therapy, the better choice both clinically and financially could be DES.

## Methods

### Study population

**Figure 2:**
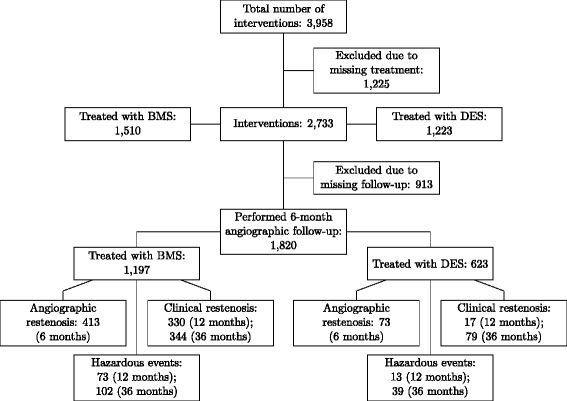
**Baseline characteristics of treatment and endpoints.** Leaves indicate endpoints for the respective patient subgroup. Endpoints refer to the respective patient subgroup, denoted by their parent node, and are not mutually exclusive. For patients treated with BMS, clinical restenosis occurred mostly during the first 12 months after intervention, whereas it occurred mostly after 12 months for patients treated with DES.

This is a retrospective observational study incorporating data from two studies at the Deutsches Herzzentrum (Munich, Germany) [28,29]. Data were originally collected between 1999 and 2006 to evaluate the prognostic value of in vitro diagnostic (IVD) biomarkers. Both studies conformed to principles of the Declaration of Helsinki and were approved by the institutional ethics committee at Deutsches Herzzentrum (Munich, Germany). All patients gave informed consent for angiographic examination and collection of in vitro diagnostic biomarkers. Based on these data sets, we performed secondary analysis to estimate treatment proportions and associated adverse effects. All subjects underwent coronary angiography due to chest pain or other symptoms suggestive of coronary artery disease (CAD). Our analyses was based on 2,733 interventions with known treatment. This data set comprised 2,377 patients of which 913 were lost to follow-up, i.e., withdrew consent or were unreachable. For the remaining 1,820 interventions, DES were used for 34.2%, and the remainder were treated with BMS (see Figure 2).

### Follow-up and endpoints

We investigated two main endpoints, restenosis and hazardous events. For restenosis, we considered angiographic restenosis or clinical restenosis. Angiographic restenosis was defined when the treated vessel developed a blockage with more than 50% diameter compared to the reference diameter proximal to the occlusion. Angiographic restenosis was evaluated at six months after the initial treatment as identified by a quantitative coronary angiography (QCA) exam. Restenosis most likely occurs in the first six months after BMS treatment [2-4]; thus, we selected this time point in our risk assessment of BMS. Clinical restenosis is based on whether the patient required an additional intervention, termed target lesion revascularization, and is typically evaluated one and three years after intervention. We included clinical restenosis to enable comparison with studies that used this specific endpoint as a proxy for angiographic restenosis.

Hazardous events were defined as a composite of three major adverse endpoints: death of any cause, myocardial infarction, and thrombosis. Particularly, hazardous events do not comprise target lesion revascularization, unlike the common clinical endpoint Major Adverse Cardiac Events (MACE). For DES we wanted to predict the risk of hazardous event, which has been shown to increase late (>1 year) after intervention [7,8]. We examined hazardous events at one and three years after intervention. All end points are summarized in Figure 2.

### Predictive variables

Our predictive models were based on a variety of features. We recorded age, sex and diabetes indication for each record, along with 30 clinical variables (including angiographic measurements) and 11 in vitro diagnostic variables, which comprised lipid panel and cardiac markers. Since data were collected over a period of seven years, not all variables could be recorded for all patients in the entire time span. Therefore, we constructed three sets of features from the complete family of clinical variables and biomarkers, respectively. Clinical variables were divided into three sets of 28, 25 and 22 features, respectively (see Table 1). IVD variables were split into one set of eight and two sets of five measurements (see Table 2). All continuous features were normalized to have zero mean and unit variance.

**Table 1:** **Predictive clinical variables**

	C1	C2	C3
AHA/AAC lesion class	+	+	+
Angina	+	+	+
Canadian cardiovascular society (CCS) grading of angina pectoris	+	+	+
Angulation	+	-	+
Body mass index	+	+	+
Hypercholesterolemia	+	+	+
Dissection	+	-	+
Ejection fraction	+	+	+
Presence of eccentric lesion	+	+	+
Family history	+	+	+
History of bypass	+	+	+
History of Intervention	+	+	+
History of myocardial infarction	+	+	+
Hypertension	+	+	+
Lesion length	+	+	+
Left ventricular function	+	+	-
Minimum lumen diameter (pre)	+	+	+
Smoker	+	+	+
Number of stents	+	+	-
New York heart association class	+	+	+
Patient height	+	+	+
Patient weight	+	+	+
Plaques	+	+	-
Reference diameter (pre)	+	+	+
ST elevation	-	+	-
Stenosis type	+	+	-
Thrombus	+	-	-
Thrombolysis in myocardial infraction (TIMI) rating	+	+	+
Tortuosity	+	-	-
Vessels affected	+	+	+

List of clinical variables that were included in the different feature sets (C1, C2, C3). Plus signs indicate that a feature has been included, minus signs that it was not included in the respective feature set.

**Table 2:** **Predictive in vitro diagnostic biomarker variables**

	B1	B2	B3
Cholesterol	+	-	-
Creatine kinase (all isoforms)	+	+	-
Creatine kinase MB isoenzyme	+	+	-
Creatinine	-	+	+
C-reactive protein	+	+	+
High density lipoprotein	+	-	-
Low density lipoprotein	+	-	-
N-terminal pro-brain natriuretic peptide	-	-	+
Triglycerides	+	-	-
Troponin T	+	+	+
high-sensitivity troponin T	-	-	+

List of in vitro diagnostic biomarkers that were included in the biomarker feature sets (B1, B2, B3). Plus signs indicate that a feature has been included, minus signs that it was not included in the respective feature set.

### Notations

Here, we consider families of features that represent different aspects of the patient’s health and/or modalities. For instance, a patient’s blood pressure, weight and disease history belongs to the family of clinical features, whereas measurements of in vitro diagnostic biomarkers comprise their own family. Each group of features derived from one family of features, is referred to as *feature set*. A classifier that considers two feature sets *i* and *j* from two different families is denoted by ${T}_k^{i,j}$, where *k* ∈ {*R*, *H*} corresponds to prediction of *P*(Restenosis|BMS) and *P*(Hazard|DES), respectively. For simplicity, we drop the indices *i* and *j* if the feature sets have been fixed in previous steps. The predicted probability of restenosis by *T*_*R*_ is denoted as *P*_*R*_ and the predicted probability of hazardous events by *T*_*H*_ as *P*_*H*_. Thresholds on the predicted probabilities are denoted as *θ*_*R*_ and *θ*_*H*_, respectively, and allow the calculation of specificity (Spec), sensitivity (Sens), positive predictive value (PPV), and negative predictive value (NPV). When applying a threshold, a classifier’s prediction can be positive (*P*_*k*_ ≥ *θ*_*k*_) or negative (*P*_*k*_ < *θ*_*k*_), which we denote by ${T}_k^{i,j}=0$ and ${T}_k^{i,j}=1$, respectively. Probability estimates of treatments and complications are denoted as $\widehat{P}\left(\cdotp \right)$ or $\overset{\sim }{P}\left(\cdotp \right)$, depending on whether estimates are based on classifiers’ performances or on amounts retrieved directly from the data set itself or from literature. For instance, the quantity $\tilde{P}\left(\mathrm{Restenosis}\left|\mathrm{sDES}\right.\right)$ denotes the probability of restenosis for patients that have been assigned DES treatment by the proposed method. It is estimated by counting the actual number of restenosis events in this patient subgroup, as determined by clinical follow-up. In contrast, $\widehat{P}\left(\mathrm{Hazard}\left|s\mathrm{D}\mathrm{E}\mathrm{S}\right.\right)$ is the estimated probability of hazardous events for the same patient subgroup and is estimated by a classifier’s ability to predict hazardous events accurately. Finally, Prev(Restenosis|BMS) denotes the prevalence of restenosis among BMS receivers in the overall population (“Treated with BMS” node in Figure 2), and Prev(Hazard|DES) represents the prevalence of hazardous events among all DES receivers in the overall population (“Treated with DES” node in Figure 2).

### Predictive modeling

**Figure 3:**
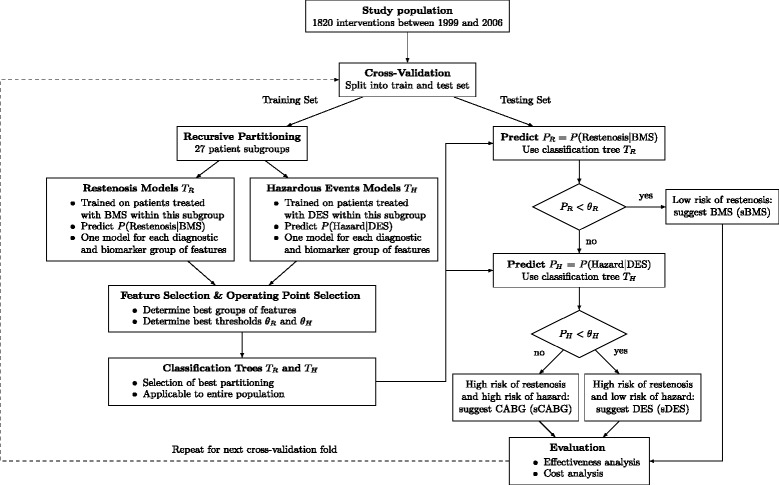
**Flowchart showing the proposed workflow.** First, we split the data set into separate training and test sets. Training starts by performing binary splits according to age, sex and diabetes indication (left). The resulting 27 patient subgroups formed the leaf nodes of multiple decision trees. Each decision tree corresponds to one way of partitioning the date set (Figure 4, left). For each patient subgroup, we were seeking two classifiers: one to assess the risk of restenosis when treated with bare-metal stents (BMS; *T*_R_) and one to assess the risk of hazardous events when treated with drug-eluting stents (DES; *T*_H_). To account for varying feature availability and feature importance among subgroups and outcomes, we considered three sets of clinical features (Table 1), three sets of in vitro diagnostic biomarkers (Table 2), as well as not using any clinical and/or biomarker features. The best set of features for each patient subgroup was determined in the feature selection step. At the same time, we chose appropriate thresholds *θ*_R_ and *θ*_H_ on the predicted probabilities of restenosis (*P*_R_) and hazardous events (*P*_H_), respectively (Figure 4, right). Training was concluded by selecting the best overall classification tree by aggregating the performances of its patient subgroup-specific models. After completing training, we applied the learned classification trees *T*_R_ and *T*_H_ on an independent test set (right). First, we used the classification tree *T*_R_ to predict *P*(Restenosis|BMS). If its results is negative (*P*_*R*_ < *θ*_*R*_), treatment with BMS is suggested, otherwise the second classification tree *T*_H_ is used to predict *P*(Hazard|DES). It suggests either DES treatment, if the predicted risk of hazardous events is low (*P*_*H*_ < *θ*_*H*_), or coronary artery bypass grafting (CABG) otherwise. Finally, we evaluated the models by estimating treatment risks and costs.

We propose a two-stage patient stratification scheme that is based on the current clinical understanding of the risks of BMS, DES and CABG treatment (outlined above). We first estimated the risk of restenosis when treated with BMS, followed by estimating the risk of hazardous events when treated with DES (see Figure 3).

If the rate of hazardous events is similar for BMS and DES [11-14], it is sufficient to estimate the risk of restenosis, when treated with BMS, and to suggest BMS only for low-restenosis-risk patients (see *T*_R_ in Figure 3). Furthermore, if DES and CABG treatment mostly differ in the rate of hazardous events [1,14], only the risk of these events needs to be estimated when treated with DES, to suggest one or the other (see *T*_H_ in Figure 3). We assumed that the risk of restenosis under BMS and the risk of hazardous events under DES are independent (*P*_R_ and *P*_H_ in Figure 3). This assumption stemmed from the fact that pathophysiologies of in-stent restenosis and in-stent thrombosis are different [30,31]. In other words, these two are competing risks and considered mutually exclusive.

Therefore, we trained two classifiers: one to predict the probability of restenosis when treated with BMS and one to predict the probability of hazardous events when treated with DES. Each of these classifiers was actually a composite of multiple patient subgroup-specific classifiers in the form of a decision tree by recursively partitioning the data set and training a logistic regression model in the leaf nodes [32]. This addresses two aspects: 1) different effects or interpretations of variables on the predicted outcome in-between these patient subgroups, and 2) a highly heterogeneous data set, which may only have a subset of features available for all patients.

### Recursive partitioning

**Figure 4:**
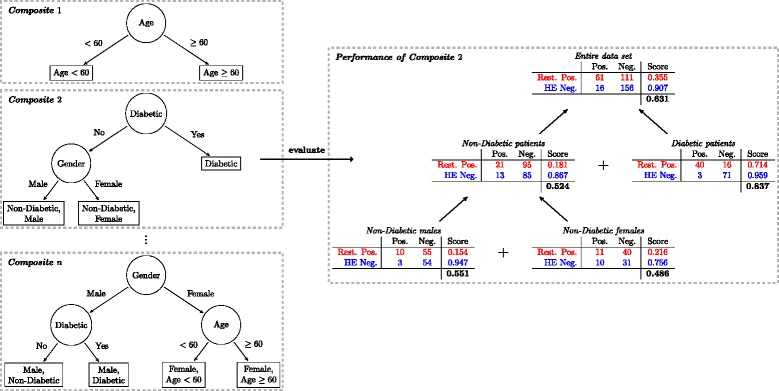
**Example of recursive partitioning and evaluation of classification trees.** First, we recursively divided the data set into patient subgroups and, for each subgroup, learned two logistic regression models for prediction of *P*(Restenosis | DES) and *P*(Hazard | DE*S*), respectively. A pair of patient-subgroup-specific classifiers is denoted as solid rectangle on the left. Next, we constructed different classification trees, corresponding to different partitionings of the data set, by combining different patient-subgroup-specific classifiers to be applicable to the entire data set (dashed boxes on the left). In total, we created and evaluated n = 47 different trees. Finally, we selected the single best performing classification tree. The performance of each classification tree was the aggregated performances of its components (dashed box on the right). We used positive predictive value for the restenosis classifiers (red) and negative predictive value for hazardous events classifiers (blue). The mean of both values denotes the overall performance (bold). In the confusion matrices on the right, the first row (red) contains true positives and false positives with respect to *P*(Restenosis | DES), and the second row (blue) false negatives and true negatives with respect to *P*(Hazard | DE*S*). Numbers do not represent actual results but only serve to illustrate our approach.

The driving hypothesis was that by stratifying the entire population into subgroups based on some clinically motivated features, we could build a better overall classifier. First, the clinical interpretation of various biomarkers could drastically differ among age groups and genders [33,34], and second, diabetes is already known to be one of the main indicators in deciding PCI treatment option [35]. Therefore, we considered these variables as the main features to recursively partition the data set into patient subgroups as shown in Figure 4. We first performed a binary split according to one feature and then performed additional splits on the resulting patient subgroups with respect to other features. The result was a decision tree whose structure depends on the order and number of binary splits. The maximum depth of a decision tree was limited to four levels, because we only split by age, gender and diabetes indication (see Table 3). We compensated for the limit in tree depth, by training regularized logistic regression models in the leave nodes. We constructed all possible trees (47 in total), with respect to the features age, sex, and diabetes indication, and selected the optimal one as explained below. This is in contrast to greedy algorithms for constructing decision trees, such as CART [32], which do not guarantee an optimal decision tree for a given set of splits.

**Table 3:** **Different combinations of features used for creating patient subgroups**

	Patient subgroups		
Age	All	≤60	>60
Sex	All	Female	Male
Diabetes	All	No diabetes	With diabetes

In total, the complete data set was partitioned into 27 different patient subgroups.

Each partitioning of the data set resulted in a decision tree with the respective patient subgroups as leaves (see Figure 4, left). At each leaf node, we consider two patient subgroups with respect to treatment options of BMS and DES, corresponding to *T*_*R*_ and *T*_*H*_ in Figure 3. Here, we split according to three features and therefore obtained 27 different patient subgroups at the leaf level across all decision trees (including trees where one or more splits have not been performed).

### Leaf node logistic regression model

For each patient subgroup, i.e., leaf node in Figure 4, we trained multiple models to predict *P*(Restenosis|BMS) and *P*(Hazard|DES), respectively. As stated in section “Predictive Variables”, training of multiple models was necessary to account for varying feature availability as well as different effects of features on the treatment options. Thus, the models trained in each subgroup differed by the sets of clinical and biomarker features they considered. Furthermore, for some combination of patient subgroup and feature sets, it might not be possible to construct a classifier due to missing data.

Let us consider *N* different sets of clinical features, *M* different sets of in vitro diagnostic biomarkers, as well as the option of not using any clinical and/or biomarkers information. Therefore, for each of 27 patient subgroups at the leaf level, we need to consider at most *NM* different combinations of clinical and biomarker feature sets – depending on data availability. For instance, with *N* = *M* = 4, the maximum number of classifiers that have to be trained is 432.

We used logistic regression with *ℓ*_2_ regularization to effectively deal with multicollinearity [36,37]. The classifiers ${T}_R^{i,j}$ were trained on patients treated with BMS, and classifiers ${T}_H^{i,j}$ on patients treated with DES, where *i* ∈ {0, …, *N*} and *j* ∈ {0, …, *M*} indicate the set of clinical and biomarkers features used by the classifier or that no features from that group were used (zero value). For building the logistic regression models with *ℓ*_2_ regularization, we used Weka version 3.6.9, which uses a quasi-Newton algorithm to search for the optimal coefficients [38].

### Feature selection and operating point selection

In the previous step, the classifiers for restenosis (${T}_R^{i,j}\Big)$ and hazardous events $\left({T}_H^{i,j}\right)$ were trained on the training set portion based on maximizing the log-likelihood of the logistic regression model [36,37] for all possible patient subgroups and feature sets independently. The next step was to select the best clinical and biomarker feature sets as well as to find the best thresholds *θ*_R_ and *θ*_H_ on the predicted probabilities of restenosis and hazardous events, respectively. We obtained classifiers *T*_*R*_ and *T*_*H*_ for each of the 27 patient subgroups (leaves in Figure 4), from which we formed the cascade depicted in Figure 3 (right).

The clinical objective was to have the least disruptions to the current trend, which is primarily toward DES utilization for all patients. *T*_R_ captures the risk of restenosis for BMS patients. By maximizing the NPV of *T*_R_, we minimized the number of patients falsely suggested to get BMS as a treatment option. The second paired test *T*_H_ captures the risk of hazardous events for patients receiving DES treatment. Maximizing the PPV of *T*_H_, in fact minimizes the number of patients falsely prescribed to be treated with DES, while they are at high risk of adverse events (under such treatment). Consequently, our clinically motivated objective function for selecting the thresholds and features for classifiers *T*_R_ and *T*_H_ is

1 $$ \underset{\mathrm{i},\mathrm{j},{\uptheta}_{\mathrm{R}},\mathrm{p},\mathrm{q},{\uptheta}_{\mathrm{H}}}{max}\mathrm{N}\mathrm{P}\mathrm{V}\left({T}_R^{i,j},{\theta}_R\right)+\mathrm{P}\mathrm{P}\mathrm{V}\left({T}_H^{p,q},{\theta}_H\right) $$

Feature selection and operating point selection were carried out concurrently by doing an exhaustive search over all possible combinations of feature sets (*i*, *j*, *p* and *q*) and thresholds (*θ*_*R*_ and *θ*_*H*_). For the thresholds, all possible values that correspond to a classifier’s specificity in the range from 5% to 95% in 5% increments were considered; excluding the trivial classifiers with 0% and 100% specificity. Also, the pair of classifiers ${T}_R^{i,j}$ and ${T}_H^{p,q}$ always considered the same patient subgroup (i.e., age group, gender and diabetes indication).

Referring to the above example with clinical and biomarkers composed of four feature sets each, at least all combinations of thresholds of both classifiers need to be evaluated per patient subgroup (2 · 19 = 38 comparisons). At the other end of the spectrum, if all feature sets are available, all possible combinations of feature sets must additionally be compared (2 · 19 · 16 = 608 combinations). During training, the exact number of comparisons may vary from subgroup to subgroup and depends on the data availability with respect to particular patient subgroups. Note that feature selection, operating point selection and pairing were only performed on the training set of each cross-validation iteration and were fixed during evaluation and predictions.

### Classification tree selection

Returning to the partitioning of the data set into patient subgroups described above, the last step of the training procedure consisted of aggregating the patient-subgroup-specific classifiers *T*_R_ and *T*_H_ to form a classification tree that is applicable to the entire patient population and choosing the best performing classification tree (Figure 4, right). Hence, the result is a classification tree that is composed of a set of regularized logistic regression models applicable to different population subsets, respectively.

To determine the best partitioning into patient subgroups, we devised a greedy algorithm that maximizes the objective criterion of equation (1) and selects the best performing decomposition of the training data set into subgroup-specific classifiers (Figure 4, right). First, the predictions of all subgroup-specific classifiers on the training set were collected; next, the resulting confusion matrix was used to calculate the performance of the classification tree according to the objective function in equation (1).

Since multiple classification trees, which correspond to alternative ways of partitioning the data set, are considered, each of them is competing for the best predictive performance. For instance, a decision tree with leaves “Male”, “Female with Age ≥ 60” and “Female with Age < 60” is competing with a decision tree that only has the split according to sex (or any other combination of splits). From all competing decompositions into different patient subgroups, we picked the decomposition that maximized the objective in equation (1), which was extracted from the root nodes of the classification trees. As a result, decision trees for *T*_R_ and *T*_H_ and their respective performances were obtained.

### Evaluation

#### Effectiveness analysis

Based on the aggregated performance results of the classification tree for restenosis and hazardous events prediction, the probability of adverse effects of the two test setup in Figure 3 was estimated. We considered the composite of restenosis and hazardous events as adverse effects, thus *P*(Adverse Effect) was defined as

2 $$ P\left(\mathrm{Adverse}\;\mathrm{Effect}\right)=P\left(\mathrm{Restenosis}\right)+P\left(\mathrm{Hazard}\right) $$

Let sBMS, sDES, sCABG denote that BMS, DES, and CABG was suggested by the two test workflow as shown on the right of Figure 3, respectively. The probabilities on the right hand side of eq. (2) can be decomposed according to the three possible treatments $\mathcal{T}=\left\{\mathrm{sBMS},\mathrm{sDES},\mathrm{sCABG}\right\}$ considered here

$$ P\left(\mathrm{Restenosis}\right)={\displaystyle \sum_{t\in T}}P(t)P\left(\mathrm{Restenosis}\Big|t\right) $$

$$ P\left(\mathrm{Hazard}\right)={\displaystyle \sum_{t\in T}}P(t)P\left(\mathrm{Hazard}\Big|t\right) $$

In both equations, we estimated the probabilities of individual treatments by taking the predictive performance of classification trees *T*_R_ and *T*_H_ into account. The first test predicts the probability *P*_R_ of restenosis when treated with BMS, and is negative if *P*_R_ is lower than threshold *θ*_*R*_, in which case BMS would be suggested. Thus, the estimate $\widehat{P}\left(\mathrm{sBMS}\right)$ can be calculated by

$$ \begin{array}{c}\hfill \widehat{P}\left(\mathrm{sBMS}\right)=P\left({P}_R<{\theta}_R\right)=P\left({T}_R=0\right)=\mathrm{Prev}\left(\mathrm{Restenosis}\left|\mathrm{B}\mathrm{M}\mathrm{S}\right.\right)\left(1 - \mathrm{S}\mathrm{ens}\left({T}_R\right)\right)\hfill \\ {}\hfill +\left(1-\mathrm{Prev}\left(\mathrm{Restenosis}\left|\mathrm{B}\mathrm{M}\mathrm{S}\right.\right)\right)\mathrm{Spec}\left({T}_R\right).\hfill \end{array} $$

The second test *T*_H_ predicts the probability of hazardous events, if treated with DES. Assuming independence between the first and second test, we obtain $\widehat{P}\left(\mathrm{sDES}\right)$ as

$$ \begin{array}{l}\widehat{P}\left(\mathrm{sDES}\right)=P\left({P}_R\ge {\theta}_R,{P}_H<{\theta}_H\right)=P\left({T}_R=1\right)P\left({T}_H=0\right)=\left(1-\widehat{P}\left(\mathrm{sBMS}\right)\right)\mathrm{Prev}\left(\mathrm{Hazard}\left|\mathrm{D}\mathrm{E}\mathrm{S}\right.\right)\left(1-\mathrm{Sens}\left({T}_H\right)\right)\\ {}+\left(1-\widehat{P}\left(\mathrm{sBMS}\right)\right)\left(1-\mathrm{Prev}\left(\mathrm{Hazard}\left|\mathrm{D}\mathrm{E}\mathrm{S}\right.\right)\right)\mathrm{Spec}\left({T}_H\right).\end{array} $$

Finally, the probability of the remaining treatment CABG was simply calculated based on the previous two probabilities.

$$ \widehat{P}\left(\mathrm{sCABG}\right)=P\left({P}_R\ge {\theta}_R,{P}_H\ge {\theta}_H\right)=1-P\left({T}_R=0\right)-P\left({T}_R=1\right)P\left({T}_H=0\right)=1-\widehat{P}\left(\mathrm{sBMS}\right)-\widehat{P}\left(\mathrm{sDES}\right) $$

Next, we estimated the conditional probabilities *P*(Restenosis|sBMS) and *P*(Hazard|sDES). These probabilities depend on the performances of *T*_R_ and *T*_H_, respectively, and were estimated by

$$ \begin{array}{l}\widehat{P}\left(\mathrm{Restenosis}\left|\mathrm{sBMS}\right.\right)=P\left(\mathrm{Restenosis}\left|{T}_R\right.=0\right)=1-\mathrm{N}\mathrm{P}\mathrm{V}\left({T}_R\right)\widehat{P}\left(\mathrm{Hazard}\left|\mathrm{sDES}\right.\right)\\ {}=P\left(\mathrm{Hazard}\left|{T}_R\right.=1,{T}_H=0\right)=1-\mathrm{N}\mathrm{P}\mathrm{V}\left({T}_H\right),\end{array} $$

where we assumed independence between the two tests in the second equation.

*P*(Hazard|sBMS) and *P*(Restenosis|sDES) depend on stratification by the first and second test, respectively. We estimated these probabilities by retrieving the corresponding label from samples classified as negative by *T*_R_, which yields $\tilde{P}\left(\mathrm{Hazard}\left|\mathrm{sBMS}\right.\right)$, and by *T*_H_, to obtain $\tilde{P}\left(\mathrm{Restenosis}\left|\mathrm{sDES}\right.\right)$.

Because our data set did not contain any information about CABG treatments, we collected estimates for *P*(Hazard|sCABG) and *P*(Restenosis|sCABG) from literature [39-50], and used 8.56% and 1.9%, respectively (see Additional file 1 for details).

### Cost analysis

To evaluate the economic impact of our method, we performed a cost analysis of the proposed two test setup. The average treatment costs of the proposed setup depends on the predicted outcome of both tests and was defined as

3 $$ \widehat{P}\left(\mathrm{sBMS}\right)\left({C}_{\mathrm{sBMS}}+{C}_{\mathrm{DAPT}}\right)+\widehat{P}\left(\mathrm{sDES}\right)\left({C}_{\mathrm{sDES}}+{C}_{\mathrm{DAPT}}\right)+\widehat{P}\left(\mathrm{sCABG}\right){C}_{\mathrm{sCABG}}, $$

where *C*_*t*_ denotes the costs for suggested treatment $t\in \mathcal{T}$ and *C*_DAPT_ the costs for one year dual anti-platelet therapy. Finally, we considered average costs per patient for any corrective procedure in the case of restenosis or hazardous events.

4 $$ \widehat{P}\left(\mathrm{Hazard}\right)\left(\frac{1}{2}\left({C}_{\mathrm{MI}}+{C}_{\mathrm{Stroke}}\right)+{C}_{\mathrm{Corrective}}\right)+\widehat{P}\left(\mathrm{Restenosis}\right){C}_{\mathrm{Corrective}}, $$

where *C*_MI_ and *C*_Stroke_ are the treatment costs for myocardial infarction and stroke, respectively, and *C*_Corrective_ the costs for any corrective procedure.

### Baseline effectiveness and costs

We compared our proposed method with the baseline workflow, where most patients are treated with DES, by estimating adverse effects and costs similar to outlined above. In the baseline workflow, all patients are treated with DES except patients with 3-vessel disease, for whom the SYNTAX trial concluded that CABG would be an appropriate alternative to PCI [1]. We analyzed a PCI population, hence we did not consider CABG exclusively; instead we considered CABG and DES by equal amounts.

The overall relative amount of patients suitable for both CABG and DES treatments was calculated as *ω* = *λP*(3 ‐ vessel disease), where *λ* ∈ [0, 1] denotes the percentage of patients with 3-vessel disease that fall into this group (see Figure 1). The estimate $\tilde{P}\left(3\hbox{-} \mathrm{vessel}\kern0.24em \mathrm{disease}\right)$ was retrieved from data, based on selected subgroups. Accordingly, the baseline initial treatment costs were calculated as follows:

$$ \left(1-\omega \right)\left({C}_{\mathrm{sDES}}+{C}_{\mathrm{DAPT}}\right)+\frac{1}{2}\omega \left({C}_{\mathrm{CABG}}+{C}_{\mathrm{sDES}}+{C}_{\mathrm{DAPT}}\right). $$

For corrective procedure costs, the probability of restenosis was estimated as the weighted sum of *P*(Restenosis|DES) and *P*(Restenosis|CABG) as

$$ \left(1-\omega \right)\tilde{P}\left(\mathrm{Restenosis}\left|\mathrm{D}\mathrm{E}\mathrm{S}\right.\right)+\frac{1}{2}\omega \left(\tilde{P}\left(\mathrm{Restenosis}\left|\mathrm{D}\mathrm{E}\mathrm{S}\right.\right)+\tilde{P}\left(\mathrm{Restenosis}\left|\mathrm{CABG}\right.\right)\right). $$

A similar formula can be obtained for the costs of hazardous events.

Finally, we estimated treatment portions and associated adverse effects by retrieving estimates for *P*(Adverse Effect) and its components directly from data, i.e., it did not depend on the performance of a classifier. We used *λ* = 0.21, which is the percentage of low complexity lesions (SYNTAX scores ≤ 22) determined by the 3-year results of the SYNTAX trial [51]. For these patients, no significant difference between CABG and PCI treatment was observed.

## Results

### Cross-validation scheme

For our analyses, we used different outcomes for the restenosis label and the hazardous events label, respectively: angiographic restenosis at 6 months after intervention, early (1-year) clinical restenosis, and late (3-year) clinical restenosis for restenosis prediction, as well as early (1-year) and late (3-year) hazardous events prediction. We used these outcomes in four different analyses: 1) we analyzed angiographic restenosis and early hazardous events, 2) we considered late hazardous events instead of the 1-year outcome, 3) we considered target lesion revascularization (clinical restenosis) as a proxy for restenosis combined with early hazardous events, and 4) we combined clinical restenosis with late hazardous events.

We required that each patient subgroup using the aforementioned sets of features to have no missing values and at least 100 samples, as well as ten samples where the class variable is positive (restenosis or hazardous events). Subgroups that did not satisfy these constraints were disregarded. Consequently, we constructed between 133 and 185 classifiers, depending on the choice of the endpoint (see Additional file 1: Table S3).

To get an estimate for the expected prediction error of our proposed method, 10-fold cross-validation was applied on our data set. Furthermore, this process was repeated three hundred times with different randomly selected cross-validation splits [52]. Referring to the number of classifiers trained on each training set from above, we trained in total between 133·10·300 and 185·10·300 different classifiers during evaluation of individual endpoints. In each iteration, performance evaluation was solely carried out on the test set, which was not used during training. In addition, this allowed us to assess the variance of the expected prediction error by means of confidence intervals, which have been derived from the percentiles of the empirical distribution of the prediction error. Instead of selecting the *ℓ*_2_ penalty of individual logistic regression models based on nested cross-validation, which would have been prohibitively expensive, we fixed the *ℓ*_2_ penalty of all models at the beginning to 0.01.

### Classification trees

**Figure 5:**
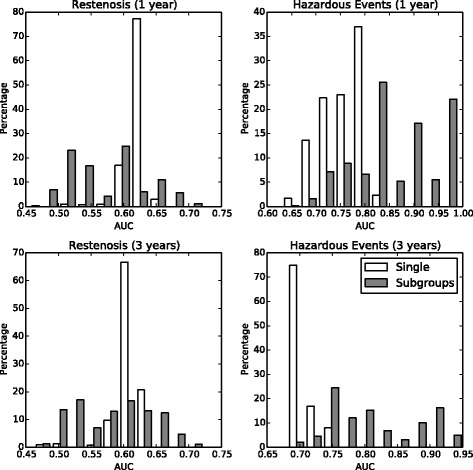
**Histograms of area under receiver operating characteristics curve for subgroup**-**specific and global classifiers.** Histograms show the area under receiver operating characteristics curve (AUC) for angiographic restenosis and hazardous events classifiers after feature selection and operating point selection. White bars indicate the performance of *ℓ*_2_ regularized logistic regression models trained on the whole population using only feature selection and operating point selection, but no partitioning. Gray bars indicate the performance of subgroup-specific classifiers.

Figure 5 compares the performance of subgroup-specific classifiers (gray bars) with conventional population wide regression (white bars). It demonstrates that prediction of hazardous events was highly discriminative with a median area under the receiver operating characteristic curve (AUC) of approximately 0.85 for the 1-year outcome and 0.75 for the 3-years outcome. Models used to predict restenosis performed modestly with a median AUC of 0.61 and 0.60. No significant differences in performance with respect to angiographic and clinical restenosis were observed. We compared the performance of complementary subgroup-specific classifiers with a single global classifier that was constructed using feature selection and operating point selection as described above, but without partitioning the data set. When predicting hazardous events, the global classifier achieved a median AUC of 0.76 and 0.71 for the 1-year and 3-years outcome. When targeting angiographic restenosis at 6 months, it resulted in an AUC of 0.57.

### Effectiveness

Table 4 summarizes the probability of adverse effects with respect to angiographic restenosis and 1-year and 3-year hazardous events, respectively. Using the two-test setup, DES was suggested for the majority of patients (81.8% at 1 year; 85.3% at 3 years). The overall probability of restenosis was 6.4% at 1 year (6.2% at 3 years) and that of hazardous events was 3.3% (7.9%). This yielded a total rate of adverse effects of 9.7% for 1-year hazardous events and 14.1% for 3-year hazardous events. Compared to the baseline treatment, where the rate of adverse effects was 14.1% and 18.8%, respectively, this resulted in a relative reduction of 31.2% (95% CI, 25.4 to 39.0) and 25.0% (95% CI, 17.8 to 30.2).

**Table 4:** **Estimates of key quantities characterizing the performance of the proposed two test workflow when 1**-**year or 3**-**year occurrences of hazardous events** (**HE**) **are taken into account along with 6**-**month angiographic restenosis results**

Estimated quantity	1- year HE		3- years HE	
	Mean	95% CI	Mean	95% CI
$\widehat{P}\left(\mathrm{sBMS}\right)$	9.7	3.6 – 18.0	5.0	2.6 – 9.5
$\widehat{P}\left(\mathrm{sDES}\right)$	81.8	74.3 – 87.5	85.3	81.4 – 87.7
$\widehat{P}\left(\mathrm{sCABG}\right)$	8.5	7.5 – 9.0	9.6	7.8 – 10.6
$\widehat{P}\left(\mathrm{Restenosis}\left|\mathrm{sBMS}\right.\right)$	0.1	0.0 – 0.0	0.0	0.0 – 0.0
$\tilde{P}\left(\mathrm{Restenosis}\left|\mathrm{sDES}\right.\right)$	7.6	6.7 – 7.9	7.0	6.1 – 7.5
$\widehat{P}\left(\mathrm{Restenosis}\right)$	6.4	5.5 – 6.9	6.2	5.3 – 6.6
$\tilde{P}\left(\mathrm{Hazard}\left|\mathrm{sBMS}\right.\right)$	0.0	0.0 – 0.0	1.5	0.0 – 16.7
$\widehat{P}\left(\mathrm{Hazard}\left|\mathrm{sDES}\right.\right)$	3.2	2.1 – 3.5	8.2	7.2 – 9.5
$\widehat{P}\left(\mathrm{Hazard}\right)$	3.3	2.5 – 3.8	7.9	6.9 – 9.5
Baseline estimate for *P*(Adverse effect)	14.1	12.6 – 14.5	18.8	17.6 – 19.6
Proposed estimate for *P*(Adverse effect)	9.7	8.3 – 10.6	14.1	12.4 – 15.6
Δ*P*(Adverse effect)	4.4	3.5 – 5.4	4.7	3.4 – 5.5

The acronyms sBMS, sDES and sCABG denote that the proposed model suggested treatment with bare-metal stents, drug-eluting stents or coronary artery bypass grafting, respectively. Baseline refers to predominant treatment with drug-eluting stents as described in section “Baseline Effectiveness and Costs”.

Similar results were obtained when analyzing clinical restenosis (see Table 5). The absolute decrease in adverse effects was highly significant with 3.2% and 4.9% (both *P* < 0.001) for the 1-year and 3-year endpoints, respectively, corresponding to 33.2% (95% CI, 26.6 to 41.9) and 25.0% (95% CI, 20.4 to 31.4) of relative reduction.

**Table 5:** **Estimates of key quantities characterizing the performance of the proposed two test workflow when 1**-**year or 3**-**year occurrences of hazardous events** (**HE**) **and clinical restenosis are taken into account**

Estimated quantity	1- year		3- years	
	Mean	95% CI	Mean	95% CI
$\widehat{P}\left(\mathrm{sBMS}\right)$	5.6	3.4 – 9.8	6.4	3.1 – 13.0
$\widehat{P}\left(\mathrm{sDES}\right)$	85.5	81.9 – 87.6	84.1	78.4 – 87.2
$\widehat{P}\left(\mathrm{sCABG}\right)$	8.9	8.2 – 9.2	9.5	8.5 – 10.4
$\widehat{P}\left(\mathrm{Restenosis}\left|\mathrm{sBMS}\right.\right)$	0.0	0.0 – 0.0	0.0	0.0 – 0.0
$\tilde{P}\left(\mathrm{Restenosis}\left|\mathrm{sDES}\right.\right)$	2.9	1.9 – 3.4	8.2	7.6 – 9.0
$\widehat{P}\left(\mathrm{Restenosis}\right)$	2.7	1.8 – 3.1	7.1	6.4 – 7.9
$\tilde{P}\left(\mathrm{Hazard}\left|\mathrm{sBMS}\right.\right)$	7.2	0.0 – 16.1	9.9	0.0 – 21.4
$\widehat{P}\left(\mathrm{Hazard}\left|\mathrm{sDES}\right.\right)$	3.2	2.0 – 3.5	7.4	5.6 – 8.9
$\widehat{P}\left(\mathrm{Hazard}\right)$	3.9	2.7 – 4.6	7.6	6.2 – 8.9
Baseline estimate for *P*(Adverse effect)	9.7	8.4 – 10.3	19.7	18.2 – 20.7
Proposed estimate for *P*(Adverse effect)	6.5	5.0 – 7.6	14.7	13.1 – 16.1
Δ*P*(Adverse effect)	3.2	2.7 – 3.8	4.9	4.1 – 6.2

The acronyms sBMS, sDES and sCABG denote that the proposed model suggested treatment with bare-metal stents, drug-eluting stents or coronary artery bypass grafting, respectively. Baseline refers to predominant treatment with drug-eluting stents as described in section “Baseline Effectiveness and Costs”.

Furthermore, we performed the same analyses without partitioning and used a single global classifier instead. With respect to angiographic restenosis, the resulting probability of adverse effects was 10.6% (95% CI, 9.1 to 12.0) and 15.8% (95% CI, 14.3 to 17.7), which is significantly higher (*P* < 0.001) compared to results above (see Additional file 1: Table S7).

### Costs

Based on the estimates $\widehat{P}\left(\mathrm{sBMS}\right)$ , $\widehat{P}\left(\mathrm{sDES}\right)$ and $\widehat{P}\left(\mathrm{sCABG}\right)$ and Medicare reimbursements in U.S. dollars of the fiscal year 2013, we calculated average costs of initial treatments and corrective procedures per patient. Thus, costs of procedures are based on the average reimbursement rates across all hospitals in the U.S., which are covered by the Medicare health insurance program (see Additional file 1 for details).

From Table 6 it is evident that the proposed setup resulted in slightly increased estimated expenses for the initial treatment ($285 to $499), but savings for any corrective procedures due to lower probability of adverse effects ($834 to $1,229). This resulted in estimated overall savings of 4.7% (95% CI, 3.1 to 7.0) and 2.7% (95% CI, 1.8 to 4.2) per patient at 1 year, when considering angiographic and clinical restenosis, respectively. Estimated total savings were equivalent to $693 and $441 per patient. Increasing the time frame to three years resulted in a modest increase of initial treatment costs. The overall costs decreased by 3.8% (95% CI, 2.7 to 5.3) and 4.0% (95% CI, 2.9 to 6.4) per patient, compared to the baseline treatment workflow. With respect to total savings, this amounted to $693 and $739, respectively.

**Table 6:** **Estimated mean costs per patient in U.S. dollar for initial treatment and corrective procedures according to equations (** 3 **) and (** 4 **), respectively**

Restenosis	Angiographic restenosis		Clinical restenosis	
Hazardous events	1-year	3-years	1-year	3-years
Initial Costs (Baseline)	13,821	13,822	13,801	13,799
Initial Costs (Proposed)	14,106	14,321	14,193	14,289
Corrective procedure (Baseline)	3,022	4,325	2,297	4,439
Corrective procedure (Proposed)	1,943	3,131	1,463	3,210
Total savings	794	693	441	739
Total savings (%)	4.71	3.82	2.74	4.05

Baseline refers to predominant treatment with drug-eluting stents as described in section “Baseline Effectiveness and Costs”.

**Figure 6:**
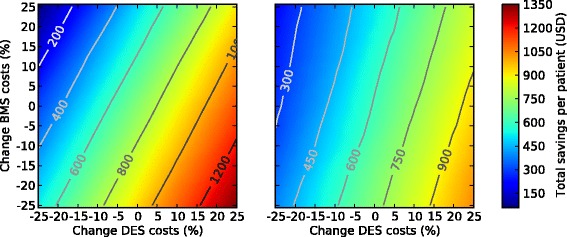
**Comparison of costs of bare**-**metal and drug**-**eluting stents with respect to total saving.** Solid lines indicate contour of total savings. Estimation is based on angiographic restenosis together with hazardous effects at 1-year (left) and at 3-years (right).

Finally, we investigated the relationship between BMS and DES procedure costs on the estimated total savings by increasing and decreasing the costs by 25% in steps of 1.25%. Therefore, BMS costs ranged between $8,900 and $14,833, and DES costs between $9,342 and $15,571. Figure 6 shows that procedure costs of BMS and DES are dependent on each other. Thus, changing the costs of one of them suggested changing the other one as well in order to sustain the same amount of savings per patient. No setting resulted in a total loss, indicating robustness to actual stent costs. Furthermore, we observed that for the 3 year time frame the cost savings decreased slightly and that the relationship between BMS and DES treatment costs was weaker (illustrated by higher slope of contour lines in Figure 6).

## Discussion

We developed a two-stage stratification procedure for patients with coronary atherosclerosis that is based on: 1) the current clinical knowledge about three common treatment options (BMS, DES, and CABG), 2) differences in feature availability and importance among patient subgroups, and 3) the objective to only deviate from the currently preferred treatment with DES for patients that would benefit – in the case of CABG – or be at no increased risk – in the case of BMS – with an alternative treatment.

We found that the proposed two-test setup resulted in an increased estimated effectiveness as well as lower estimated costs when compared to the baseline workflow in all settings. By optimizing negative and positive predictive values of patient subgroup-specific classifiers, we accounted for current clinical evidence that DES are dominating BMS only for a subset of patients [15] and that DES are a viable alternative to CABG for certain patient subgroups [1].

In the analyses of clinical restenosis, the rate of restenosis differed considerably between the 1-year and 3-year analysis. This increase is due to the fact that the time point for restenosis varied (1 or 3 years) whereas it was fixed previously (6 months). Therefore, an increase in the restenosis rate was expected due to the longer time period considered.

The current clinical practice for coronary revascularization focuses to a large extent only on PCI with DES and CABG as its alternative, but ignores treatment with BMS. However, patients at low risk of restenosis could still benefit from BMS [15]. We identified these patients and were able to minimize the estimated rate of restenosis for these patients. For a preponderating portion of validation runs the estimate $\widehat{P}\left(\mathrm{Restenosis}\left|\mathrm{sBMS}\right.\right)$ was zero. We attributed this result to the fact that both the estimate $\widehat{P}\left(\mathrm{Restenosis}\left|\mathrm{sBMS}\right.\right)$ as well as the objective in eq. (1) incorporate NPV(*T*_*R*_, *θ*_*R*_) and thus classifiers with a negative predictive value of 1 were selected preferably, which resulted in a restenosis rate of zero.

Furthermore, the SYNTAX trial [1] demonstrated that there exists a grey area where the outcome of DES and CABG treatment does not differ significantly. Hence, for some patients, PCI with DES would be as effective as CABG, but significantly less perilous. We incorporated this knowledge in our method and observed that the estimated proportion of hazardous events for patients treated with DES was low (3.2 to 8.2%).

The proposed two stage setup deviated from the baseline workflow only for a small set of patients. This is where there is no increase in risks, as in the case of BMS versus DES, or where it is very likely that they would benefit from an alternative treatment, as in the case of CABG versus DES. When evaluating treatment risks, only those factors that are most decisive – based on results of clinical studies [1,11-14] – were considered, instead of trying to model all possible risks and their interrelationships. For instance, when assessing the risks of BMS treatment we do not suggest ignoring hazardous events in this setting, but based on previous results [11-13], it is known that the rate of hazardous events differs only slightly among BMS and DES treatments. Therefore, predicting hazardous events in this setting would add little additional information.

With respect to economic implications, we noticed that average costs per patient for index procedures slightly increased, whereas average costs per patient for corrective procedures decreased. The former can be explained by the small cost difference between BMS and DES treatment ($590), together with high expenses associated with CABG ($28,683). Therefore, performing stenting twice, once for the index procedure and once for the corrective procedure, instead of one surgery would still be less expensive at the population level than treating the patient by CABG first. This severely diminished the amount of total savings, especially in early stages.

Regarding angiographic restenosis, we observed that the total amount of savings decreased from the 1-year to the 3-year hazardous events analysis and that the percentage of suggested BMS treatment dropped from 8.8% to 4.4%, favoring DES and CABG. Treating a large number of patients with procedures that were more expensive than BMS increased initial treatment costs and ultimately lowered the total savings.

It is important to note that the method proposed here and the SYNTAX score [53] – an algorithm to grade the complexity of CAD based on angiographic data – address different aspects related to CAD. Sianos et al. [53] state the SYNTAX score “is focusing on anatomy of coronary vasculature and not on treatment plan” (p. 226). The SYNTAX score II aimed to overcome this limitation by considering clinical variables in addition to the anatomical SYNTAX score. Although this model performs well, it was derived from a data set composed of patients with left main coronary disease or three-vessel disease. Thus, the SYNTAX score II can facilitate decision making only for a subset of patients that satisfy these criteria.

In addition, only lesions with more than 50% reduction in luminal diameter should be included in the SYNTAX score. In contrast, our approach does not have any exclusion criteria and is based on all available information for one patient – including those mentioned above – to assess the risk of particular treatment options. In fact, the SYNTAX score could complement our approach as an additional source of information in helping to estimate risks of therapeutic options.

These results showed that considering BMS and CABG as alternatives to DES is one effort to a more personalized treatment regime that could also positively impact treatment costs. Further research, in particular in the form of a prospective study, would provide additional insight into the magnitude of these effects.

### Limitations

One of the major limitations, subject to all retrospective observational analyses, is that treatment was determined by physicians and was not random. This inherent and unavoidable bias might lead to identifying false estimators. Because data were collected over a period of eight years, changes in technology during this time span, such as changes in data collection protocol, might have influenced obtained measurements. Therefore, temporal latent effects could possibly affect our models.

Due to the requirement of angiographic follow-up at 6 months after intervention and the collection of biomarkers, the patient population used was relatively small. As a consequence, some subgroups could not be analyzed. In our analyses, we treated multiple consecutive interventions per patient as independent and did not take the outcome of previous procedures into account. Risk prediction was formulated as a binary classification problem instead of predicting time-to-event. Furthermore, our data did not include medications and their influence on biomarker measurements could not be quantified. Finally, our estimates with respect CABG are solely based on data we retrieved from literature and models used for restenosis prediction only showed modest performance, which was observed by Amin et al. [19], too.

## Conclusions

We demonstrated that by considering multiple treatment options for coronary atherosclerosis and modeling their respective risks, the estimated overall adverse effects and costs could be reduced. This could lead to more efficacious and personalized treatment for patients, and significant economic impact at the population level. When projecting our results on the estimated 1,085,357 coronary revascularization procedures performed on adults in the U.S. in 2007 [54], this would positively affect approximately 47,756 and 51,012 patients for angiographic restenosis at one and three years, respectively, which accumulates to estimated cost savings ranging from $475.4 to $842.2 million dollars, depending on the respective outcome (angiographic or clinical restenosis) and time frame (one or three years).

### Endnotes

^a^Derived from the study of the same name (SYNergy between Percutaneous Coronary Intervention with TAXus and Cardiac Surgery).

## Additional file

Additional file 1: **Supplementary material.** PDF document containing supplementary materials.

## Abbreviations

AHA/ACC: American Heart Association/American College of Cardiology
AUC: Area under the receiver operating characteristic curve
B1-3: Feature set 1, 2, or 3 comprising in vitro diagnostic biomarkers (see Table 2)
BMS: Bare-metal stent treatment
C1-3: Feature set 1, 2 or 3 comprising diagnostic variables (see Table 1)
CABG: Coronary artery bypass grafting surgery
CAD: Coronary artery disease
CCS: Canadian Cardiovascular Society
DAPT: Dual anti-platelet therapy
DES: Drug-eluting stent treatment
HE: Hazardous events
IVD: In vitro diagnostic
MACE: Major adverse cardiac events
MI: Myocardial infarction
NPV: Negative predictive value
PCI: Percutaneous coronary intervention
$\widehat{P}\left(\cdot \right)$: Probability estimate of treatment or event given in brackets derived from classifiers’ performances
$\widehat{P}\left(\cdot \right)$: Probability estimate of treatment or event given in brackets derived from the data set itself
*P*_*H*_: Predicted probability *P*(Hazard | DES) by classifier trained on patients treated with drug-eluting stents
*P*_*R*_: Predicted probability *P*(Restenosis | DES) by classifier trained on patients treated with bare-metal stents
Prev(·): Prevalence of event given in brackets
PPV: Positive predictive value
QCA: Quantitative coronary angiography ROC, Receiver operating characteristic
sBMS: Bare-metal stent treatment suggested by the proposed workflow
sCABG: Coronary artery bypass grafting surgery suggested by the proposed workflow
sDES: Drug-eluting stent treatment suggested by the proposed workflow
Sens: Sensitivity
Spec: Specificity
SYNTAX: SYNergy between Percutaneous Coronary Intervention with TAXus and Cardiac Surgery
*θ*_*H*_: Threshold on predicted probabilities *P*_*H*_ of hazardous events
*θ*_*R*_: Threshold on predicted probabilities *P*_*R*_ of restenosis
*T*_*H*_: Classifier trained on patients treated with drug-eluting stents to predict hazardous events
*T*_*R*_: Classifier trained on patients treated with bare-metal stents to predict restenosis
TIMI: Thrombolysis In Myocardial Infraction
TVR: Target-vessel revascularization
U.S: United States
