# Supplementary material for: Stratification of coronary artery disease patients for revascularization procedure based on estimating adverse effects
Source: BMC Med Inform Decis Mak. 2015 Feb 14;15:9. doi: 10.1186/s12911-015-0131-0 (PMC4336731; doi:10.1186/s12911-015-0131-0)
Supplement: Supplementary file 1 — Supplementary material. PDF document containing supplementary materials. [file 12911_2015_131_MOESM1_ESM.pdf]

Supplement to Stratification of Coronary Artery Disease Patients for Revascularization Procedure Based on Estimating Adverse Effects

Sebastian Pölsterl, Maneesh Singh, Amin Katouzian, Nassir Navab, Adnan Kastrati, Lance Ladic, and Ali Kamen

November 27, 2014

1 Patient Characteristics

Variable	Included	Excluded	P-value
Age	65.3 ± 10.3	68.9 ± 11.3	$5.23 \cdot 10^{-15}$
Female	405 (22.3%)	255 (27.9%)	$1.26 \cdot 10^{-3}$
Diabetic	418 (23.2%)	267 (29.5%)	$4.36 \cdot 10^{-4}$

Table S1: Comparison of patient characteristics of included and excluded interventions. From a total of 2,733 interventions, we excluded 913 interventions due to missing angiographic follow-up. Categorical variables were summarized by absolute counts and relative proportions and were compared with Fisher's exact test. Continuous variables were summarized by mean and standard deviation and compared using Welch's t-test.

2 Adverse Effects for Coronary Artery Bypass Grafting

Adverse effects of CABG treatment were retrieved from previously published randomized controlled trials. The probability of hazardous events (HE) was retrieved from eleven different trials shown in table S2. The definition of restenosis in the case of CABG is not straightforward, it could refer to reduced patency in the grafted vessel due to atherosclerosis or to a previously untreated lesion upstream of the original lesion. Here, we assumed the first definition and estimated $P(\text{Restenosis}|\text{CABG})$ to be 1.9% [4].

Study	Patients	Death	MI	Ref.
ARTS	603	17	28	[5]
ERACI II	225	16.875	14.175	[6]
AWESOME	232	48.72		[7]
SoS	500	4	34	[8]
MASS II	203	5	2	[9]
Myoprotect	21	5	0	[10]
Octostent	142	4	7	[11]
AMIST	47	1	0	[12]
Cisowski et al.	24	0	0	[13]
Kim et al.	50	4		[14]
SYNTAX	849	30	28	[15]
Hong et al.	68	0	5	[16]

Table S2: Randomized Control Studies used to estimate $P(\text{Hazard}|\text{CABG})$. Numbers indicate total number of patients with at least one occurrence of the specified clinical event up to one year after intervention. Decimal numbers occur when only percentage values were provided. MI indicates myocardial infarction.

3 Subgroups

Due to a smaller amount of data available for angiographic restenosis, a smaller number of subgroups was used, compared to the analyses considering clinical restenosis. Owing to insufficient data, our evaluation did not cover females younger than 60 years of age. In addition, for endpoints after 1 year, we could not factor in diabetics younger than 60 years old, and for angiographic restenosis we could not account for diabetic females older than 60 years of age. However, patients specific to these subgroups were part of supersets of these subgroups and therefore still included in the analyses, for instance females younger than 60 years are part of females of any age.

		1-yr HE		3-yr HE	
		BMS	DES	BMS	DES
Restenosis	Angiographic	133	157	133	185
	Clinical	148	157	148	185

Table S3: Number of subgroups generated for four different analyses. Bare-metal stent subgroups (BMS) were used to train classifiers to predict the probability of restenosis, and drug-eluting stent subgroups (DES) were used to predict hazardous events (HE).

Age	Gender	Diabetes
Age ≤ 60	*	Diabetic only
Age ≤ 60	Female	*
Age > 60	Female	Diabetic only

Table S4: Excluded subgroups due to insufficient data for prediction of hazardous events at 1 year. In total 10 subgroups were excluded. Asterisks indicate all three subgroups for this particular group, e.g. all genders, male and female.

Age	Gender	Diabetes
Age ≤ 60	*	Diabetic only
Age ≤ 60	Female	*

Table S5: Excluded subgroups due to insufficient data for prediction of angiographic and clinical restenosis, and 3-year hazardous events. In total 6 subgroups were excluded. Asterisks indicate all three subgroups for this particular group, e.g. all genders, male and female.

4 Costs

The costs were estimated based on Medicare reimbursements to hospitals in the U.S. for the year 2013[1]. Costs are based on full update national adjusted operating standardized amounts and capital standard federal rate averaged across all urban and rural areas. The base payment rate was $5,549.85. Costs for CABG were calculated by taking the weighted average of six different diagnosis-related groups (DRG) based on the number of cases in 2012: 231 (1.78%), 232 (1.54%), 233 (20.13%), 234 (32.19%), 235 (12.70%), and 236 (31.66%). Costs for BMS were calculated by the weighted average of DRG 248 (28.40%) and DRG 249 (71.60%). Accordingly, for DES where DRG 246 (21.74%) and DRG 247 (78.26%) were used. Costs for myocardial infarction are based on DRG 280 (46.78%), DRG 281 (32.39%), and DRG 282 (20.83%). Finally, for stroke we used DRG 61 (32.98%), DRG 62 (51.95%), and DRG 63 (15.06%). Corrective procedure costs where estimated by taking the weighted average of all DRGs comprising CABG, BMS, and DES from above. Following the current guidelines, we assumed one year of dual anti-platelet therapy for both BMS and DES and estimated the costs as $1 per day for generic clopidogrel [2, 3]. The final costs are in 2013 U.S. dollar are shown in table S6.

Variable	Costs in USD
C_{sBMS}	11,866.11
C_{sDES}	12,456.51
C_{sCABG}	28,683.40
$C_{\text{Corrective}}$	16,668.56
C_{MI}	7,537.75
C_{Stroke}	12,169.54
C_{DAPT}	365

Table S6: Costs in 2013 U.S. dollars assigned to individual treatments.

5 Analysis without Partitioning

	1-year Hazardous Events		3-year Hazardous Events	
	Mean	95% CI	Mean	95% CI
$\hat{P}$(sBMS)	2.3	1.8 – 9.1	3.1	1.8 – 9.1
$\hat{P}$(sDES)	90.5	84.8 – 92.0	89.1	83.3 – 90.5
$\hat{P}$(sCABG)	6.5	5.7 – 7.0	7.9	7.1 – 8.2
$\hat{P}$(Restenosis|sBMS)	0.0	0.0 – 0.0	0.0	0.0 – 0.0
$\tilde{P}$(Restenosis|sDES)	6.6	6.2 – 6.8	6.9	6.7 – 7.2
$\hat{P}$(Restenosis)	6.2	5.5 – 6.4	6.3	5.8 – 6.7
$\tilde{P}$(Hazard|sBMS)	0.2	0.0 – 0.0	4.5	0.0 – 33.3
$\hat{P}$(Hazard|sDES)	4.4	3.6 – 5.6	9.7	7.4 – 10.2
$\hat{P}$(Hazard)	4.5	3.6 – 5.6	9.5	7.4 – 11.7
Baseline P(Adverse Effect)	12.3	11.2 – 13.4	18.6	17.4 – 18.8
Proposed P(Adverse Effect)	10.6	9.1 – 12.0	15.8	14.3 – 17.7
ΔP(Adverse Effect)	1.7	1.4 – 2.6	2.8	0.9 – 3.8
Relative Risk Reduction (%)	13.8	10.2 – 20.1	15.1	4.7 – 20.5
Estimated Costs				
Initial Costs (Baseline)	13,795	13,749 – 13,847	13,822	13,782 – 13,852
Initial Costs (Proposed)	13,829	13,674 – 13,923	14,052	13,897 – 14,116
Corrective procedure (Baseline)	2,650	2,380 – 2,910	4,297	3,871 – 4,347
Corrective procedure (Proposed)	2,221	1,858 – 2,560	3,579	3,101 – 4,094
Total Savings	395	242 – 742	487	132 – 768
Total Savings (%)	2.4	1.5 – 4.4	2.7	0.7 – 4.2

Table S7: Estimated probabilities of adverse effects for angiographic restenosis and 1-year and 3-year hazardous events analysis, respectively, without constructing subgroup-specific classifiers. Baseline refers to treatment exclusively with drug-eluting stents and costs are in 2013 U.S. dollar.

References

[1] Department of Health and Human Services. *Federal Register Volume 77, Issue 170 (August 31, 2012)*, chapter Rules

and Regulations, pages 53257–53750. Office of the Federal Register, National Archives and Records Administration, 2012.

[2] Mahoney EM, Wang K, Arnold SV, *et al.* Cost-effectiveness of prasugrel versus clopidogrel in patients with acute coronary syndromes and planned percutaneous coronary intervention: Results from the trial to assess improvement in therapeutic outcomes by optimizing platelet inhibition with prasugrel–thrombolysis in myocardial infarction triton-timi 38. *Circulation* 2010; **121**(1):71–79.

[3] Levine GN, Bates ER, Blankenship JC, *et al.* 2011 ACCF/AHA/SCAI Guideline for Percutaneous Coronary Intervention. A report of the American College of Cardiology Foundation/American Heart Association Task Force on Practice Guidelines and the Society for Cardiovascular Angiography and Interventions. *J Am Coll Cardiol* Dec 2011; **58**(24):e44–122.

[4] Singh RN. Atherosclerosis and the internal mammary arteries. *Cardiovasc Intervent Radiol* 1983; **6**(2):72–77.

[5] Serruys PW, Ong ATL, van Herwerden LA, *et al.* Five-Year Outcomes After Coronary Stenting Versus Bypass Surgery for the Treatment of Multivessel DiseaseThe Final Analysis of the Arterial Revascularization Therapies Study (ARTS) Randomized Trial. *J Am Coll Cardiol* Aug 2005; **46**(4):575–581.

[6] Rodriguez A, Bernardi V, Navia J, *et al.* Argentine randomized study: coronary angioplasty with stenting versus coronary bypass surgery in patients with multiple-vessel disease (ERACI II): 30-day and one-year follow-up results. *J Am Coll Cardiol* Jan 2001; **37**(1):51–58.

[7] Morrison DA, Sethi G, Sacks J, *et al.* Percutaneous coronary intervention versus coronary artery bypass graft surgery for patients with medically refractory myocardial ischemia and risk factors for adverse outcomes with bypass: a multicenter, randomized trial. *J Am Coll Cardiol* Jul 2001; **38**(1):143–149.

[8] Investigators TS. Coronary artery bypass surgery versus percutaneous coronary intervention with stent implantation in patients with multivessel coronary artery disease (the Stent or Surgery trial): a randomised controlled trial. *Lancet* Sep 2002; **360**(9338):965–970.

[9] Hueb W, Soares PR, Gersh BJ, *et al.* The medicine, angioplasty, or surgery study (MASS-II): a randomized, controlled clinical trial of three therapeutic strategies for multivessel coronary artery diseaseOne-year results. *J Am Coll Cardiol* May 2004; **43**(10):1743–1751.

[10] Pohl T, Giehrl W, Reichart B, *et al.* Retroinfusion-supported stenting in high-risk patients for percutaneous intervention and bypass surgery: Results of the prospective randomized myoprotect I study. *Catheter Cardiovasc Interv* 2004; **62**(3):323–330.

[11] Eefting F, Nathoe H, van Dijk D, *et al.* Randomized Comparison Between Stenting and Off-Pump Bypass Surgery in Patients Referred for Angioplasty. *Circulation* Dec 2003; **108**(23):2870–2876.

[12] Reeves BC, Angelini GD, Bryan AJ, *et al.* A multi-centre randomised controlled trial of minimally invasive direct coronary bypass grafting versus percutaneous transluminal coronary angioplasty with stenting for proximal stenosis of the left anterior descending coronary artery. *Health Technol Assess* Apr 2004; **8**(16):1–43.

[13] Cisowski M, Drzewiecki J, Drzewiecka-Gerber A, *et al.* Primary stenting versus MIDCAB: preliminary report–Comparision of two methods of revascularization in single left anterior descending coronary artery stenosis. *Ann Thorac Surg* Oct 2002; **74**(4):1334–1339.

[14] Kim JW, Lim DS, Sun K, *et al.* Stenting or MIDCAB using ministernotomy for revascularization of proximal left anterior descending artery? *Int J Cardiol* Mar 2005; **99**(3):437–441.

[15] Serruys PW, Morice MC, Kappetein AP, *et al.* Percutaneous Coronary Intervention versus Coronary-Artery Bypass Grafting for Severe Coronary Artery Disease. *N Engl J Med* Mar 2009; **360**(10):961–972.

[16] Hong SJ, Lim DS, Seo HS, *et al.* Percutaneous coronary intervention with drug-eluting stent implantation vs. minimally invasive direct coronary artery bypass (MIDCAB) in patients with left anterior descending coronary artery stenosis. *Catheter Cardiovasc Interv* 2005; **64**(1):75–81.
